# The Prognostic Roles of Pretreatment Circulating Tumor Cells, Circulating Cancer Stem-Like Cells, and Programmed Cell Death-1 Expression on Peripheral Lymphocytes in Patients with Initially Unresectable, Recurrent or Metastatic Head and Neck Cancer: An Exploratory Study of Three Biomarkers in One-time Blood Drawing

**DOI:** 10.3390/cancers11040540

**Published:** 2019-04-15

**Authors:** Pei-Hung Chang, Min-Hsien Wu, Sen-Yu Liu, Hung-Ming Wang, Wen-Kuan Huang, Chun-Ta Liao, Tzu-Chen Yen, Shu-Hang Ng, Jen-Shi Chen, Yung-Chang Lin, Hung-Chih Lin, Jason Chia-Hsun Hsieh

**Affiliations:** 1 Chang Gung University, College of Medicine, Taoyuan 333, Taiwan; ph555chang@cgmh.org.tw (P.-H.C.); n820903@gmail.com (S.-Y.L.); whm526@adm.cgmh.org.tw (H.-M.W.); medfoxtaiwan@gmail.com (W.-K.H.); Liaoct@adm.cgmh.org.tw (C.-T.L.); Yen1110@adm.cgmh.org.tw (T.-C.Y.); shng@adm.cgmh.org.tw (S.-H.N.); js1101@cgmh.org.tw (J.-S.C.); yclinof@cgmh.org.tw (Y.-C.L.); sangerhj@gmail.com (H.-C.L.); 2 Division of Hematology-Oncology, Department of Internal Medicine, Chang Gung Memorial Hospital, Keelung 20401, Taiwan; 3 Cancer Center, Chang Gung Memorial Hospital, Keelung 20401, Taiwan; 4 Circulating Tumor Cell Lab, Division of Hematology-Oncology, Department of Internal Medicine, Chang Gung Memorial Hospital, Linkuo 333, Taiwan; mhwu@mail.cgu.edu.tw; 5 Graduate Institute of Biochemical and Biomedical Engineering, Chang Gung University, Taoyuan 333, Taiwan; 6 Department of Chemical Engineering, Ming Chi University of Technology, New Taipei City 24301, Taiwan; 7 Department of Oncology–Pathology, Karolinska Institutet, Stockholm, Sweden; Cancer Center Karolinska, Karolinska University Hospital, SE-17176 Stockholm, Sweden; 8 Department of Otorhinolaryngology, Head and Neck Surgery, Chang Gung Memorial Hospital and Chang Gung University, Taoyuan 333, Taiwan; 9 Molecular Imaging Center, Linkou Chang Gung Memorial Hospital and Chang Gung University, Taoyuan 333, Taiwan; 10 Department of Nuclear Medicine, Linkou Chang Gung Memorial Hospital and Chang Gung University, Taoyuan 333, Taiwan; 11 Department of Diagnostic Radiology, Linkou Chang Gung Memorial Hospital and Chang Gung University, Taoyuan 333, Taiwan; 12 Department of Medical Imaging and Radiological Sciences, Chang Gung Memorial Hospital, Chang Gung University, Taoyuan 333, Taiwan

**Keywords:** circulating tumor cells, cancer stem-like cells, PD-1, peripheral lymphocytes, head and neck cancer

## Abstract

Circulating tumor cells (CTCs) and immune status are strongly related to cancer prognosis, although few studies have examined both factors. This prospective observational study (ClinicalTrials.gov: NCT02420600) evaluated whether CTCs, circulating cancer stem-like cells (cCSCs), and peripheral lymphocytes with/without Programmed cell death protein 1 (PD-1) expression were associated with prognosis among patients receiving palliative chemotherapy for initially unresectable, recurrent/metastatic head and neck squamous cell carcinoma (rmHNSCC). Thirty-four patients were enrolled between January 2015 and June 2016. Overall survival (OS) was associated with a higher CTC number (hazard ratio [HR]: 1.01, *p* = 0.0004) and cCSC ratio (HR: 29.903, *p* < 0.0001). Progression-free survival (PFS) was also associated with CTC number (HR: 1.013, *p* = 0.002) and cCSC ratio (HR: 10.92, *p* = 0.003). A CD8^+^ proportion of ≥ 17% was associated with improved OS (HR: 0.242, *p* = 0.004). A CD4: CD8 ratio of >1.2 was associated with poorer trend of PFS (HR: 2.12, *p* = 0.064). PD-1 expression was not associated with survival outcomes. Baseline CTCs, cCSC ratio, and CD8^+^ ratio may predict prognosis in rmHNSCC.

## 1. Introduction

There are >500,000 new cases of head and neck squamous cell carcinoma (HNSCC) annually, with approximately 400,000 deaths worldwide [1,2]. Furthermore, the rates of new HNSCC cases and its incidence are 60,000 cases/year and 11.0/100,000 population in the USA, 63,500 cases/year and 11.2/100,000 population in Europe, and 7200 cases/year and 20.72/100,000 population in Taiwan [3,4]. Chemotherapy with or without targeted therapy remains a standard treatment for patients with initially unresectable, recurrent or metastatic HNSCC, although salvage surgery and re-irradiation may be possible for potentially resectable cases [5]. However, survival after disease progression or distant metastasis is relatively short, compared to other cancers [6], which contributes to the limited ability to switch chemotherapies. Moreover, the lack of useful and validated biomarkers to predict response is a major challenge during front-line palliative chemotherapy.

One promising biomarker is circulating tumor cells (CTCs), which can predict prognosis before starting therapy for various types of cancer [7,8,9]. These CTCs are shed from the primary tumor and can be detected in the patient’s bloodstream, where they often express epithelial markers, such as epithelial cell adhesion molecule (EpCAM) [10] or cytokeratins [11], but typically do not express white and red blood cell markers, such as CD45 [12] and CD235a [13]. One exception is CTCs that are undergoing epithelial-mesenchymal transition processes [14]. Many investigators have found that CTCs can predict treatment response and have proposed monitoring CTC counts during anticancer therapy [15]. Moreover, a small subset of CTCs expresses stem cell markers, such as CD133 [16] or CD44 [17]. These cells can be referred to as circulating cancer stem-like cells (cCSCs), and are thought to be partially related to chemotherapy resistance [18,19,20]. Kantara et al. have reported several methods for identifying cCSCs using their expression of DCLK1, Lgr5, ANXA2, and PG, rather than CD44, and found that cCSCs were consistently related to chemotherapy resistance [21]. Nevertheless, the role of cCSCs in HNSCC remains unclear, especially regarding whether they are associated with prognosis or chemoresistance.

The relationship between host immune status and cancer cells is incompletely understood, and there has been a significant recent interest in the interaction between cancer and the host’s immune status (e.g., CD4^+^ count, CD8^+^ count, CD4: CD8 ratio, and CD56^+^ fraction of peripheral lymphocytes) [22,23,24,25,26,27]. Programmed cell death protein 1 (PD-1) is a 55-kDa transmembrane protein with one extracellular IgV-like domain and a 97-amino acid cytoplasmic tail consisting of one immunotyrosine switch motif and one immunotyrosine inhibitory motif. PD-1 can be expressed on activated T cells, B cells, and myeloid cells. The function of PD-1 has been revealed to be an inhibitory role and a potential role in regulating tolerance and autoimmunity. PD-L1 is commonly upregulated on many different tumor types, where it inhibits anti-tumor T cell responses, and that PD-1 is expressed on the majority of tumor-infiltrating lymphocytes That is an important rationale to block this pathway as novel cancer immunotherapies. One study has revealed that PD-1 expression on lymphocytes is significantly correlated with cancer prognosis. [22] For example, PD-1 expression on peripheral CD4^+^ lymphocytes reflects impaired T-cell function and predicts poor clinical outcomes [23,24].

However, no studies have examined the associations of survival with PD-1 expression on peripheral lymphocytes (i.e., host immune status) plus the patient’s CTC and cCSC status (i.e., markers for cancer aggressiveness). Therefore, this study aimed to evaluate whether CTCs, cCSCs and peripheral lymphocytes with/without PD-1 expression were associated with prognosis and therapy response among patients who were receiving palliative chemotherapy for initially unresectable, recurrent/metastatic HNSCC.

## 2. Results

### 2.1. Patient Enrollment

**Figure 1:**
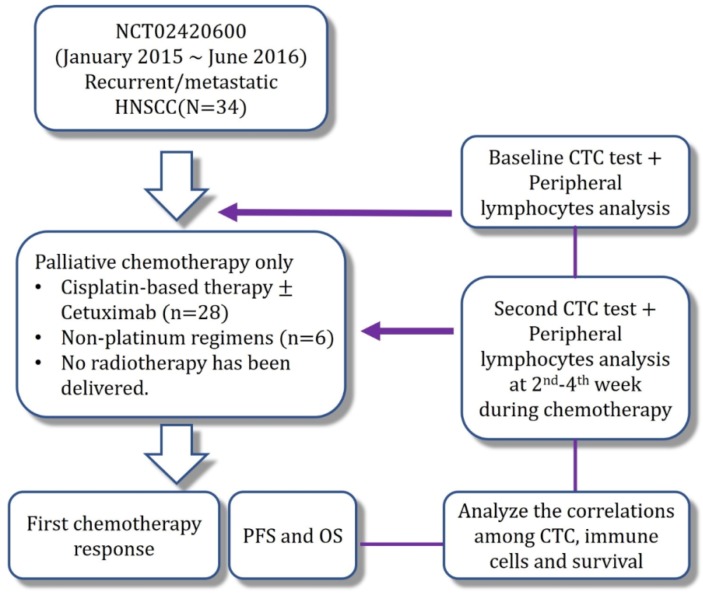
Study flow chart.

Between January 2015 and June 2016, 34 patients were enrolled after receiving a detailed explanation regarding the study’s design, methods, and potential risks (Figure 1). The patients’ basic characteristics are shown in Table 1, and all patients were followed until September 2017. The median follow-up time was 5.6 months ranging from 1.0–29.0 months. The median patient age was 50 years (range: 37–73 years), and most patients were male (85.3%). The most common primary sites were the oral cavity (55.9%) and oropharynx (23.5%). Most patients had an Eastern Cooperative Oncology Group performance status of 0–1 (61.4%), and a smaller subgroup had a performance status of ≥2 (38.6%). The cases were classified as initially unresectable or recurrent (*n* = 4) or metastatic (*n* = 30), and no radiation was delivered during this study, although all patients received ≥2 full cycles of palliative chemotherapy. The most common sites of distant metastasis were the lungs (53.3%) and distant lymph nodes or soft tissue metastasis (36.7%). The chemotherapy regimens mainly involved cisplatin-based regimens with or without cetuximab (82.4%). Not all patients were evaluated for p16 expression, which was only detected in 8.8% (3/34) of the total population and 27.3% (3/11) in evaluated cases.

### 2.2. Multivariate Analysis of Survival Outcomes

**Figure 2:**
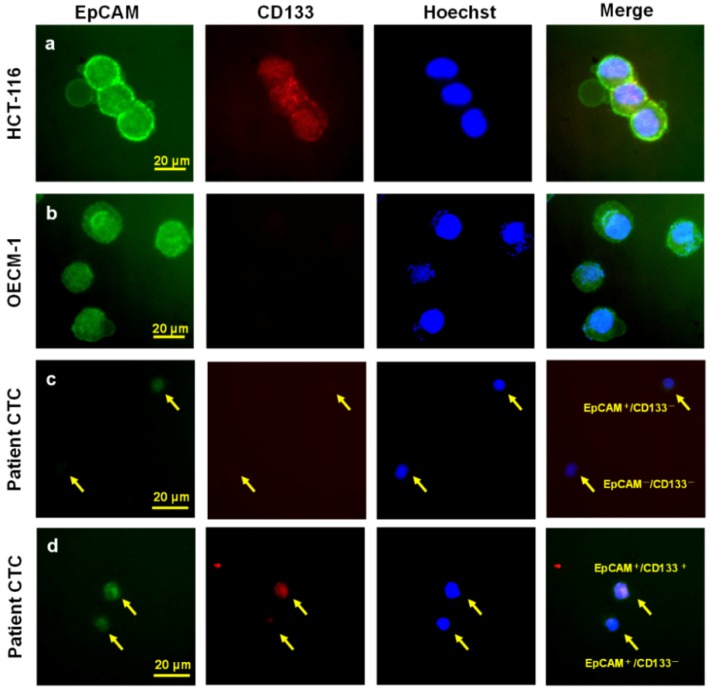
Detecting circulating tumor cells (CTCs) and circulating cancer stem-like cells (cCSCs) using immunofluorescence (Patient No.16 and Patient No.21). (**a**) HCT116 as the demonstrated cell line for EpCAM^+^/CD133^+^ cells. (**b**) The OECM-1 as the demonstrated cell line for EpCAM^+^/CD133^−^ cells. (**c**) In Patient No. 16, the image showed the EpCAM^+^/CD133^−^ and EpCAM^−^/CD133^−^ CTCs. (**d**) In Patient No. 21, the image showed the EpCAM^+^/CD133^+^ and EpCAM^+^/CD133^−^ CTCs.

**Figure 3:**
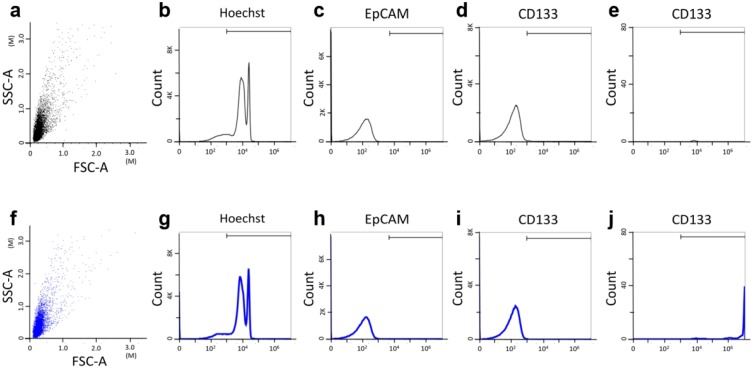
Detecting circulating tumor cells (CTCs) and circulating cancer stem-like cells (cCSCs) using flow cytometry. Panel (**a**–**e**) shows the isotype control (Hoechst and mouse IgG isotypes for EpCAM and CD133) for CTC and cCSC analysis. Panel (**f**–**j**) demonstrates real cancer patients’ CTC and cCSC by staining with Hoechst, EpCAM, and CD133 antibodies after red and white blood cells depletion protocol. The gating algorithms are SSC and FSC for whole cell distribution in panel (**a**,**f**). Then we gated cells with positive Hoechst expression nucleated cells population in panel (**b**,**g**). Based on positive Hoechst situation, EpCAM positive cells were selected and defined as CTCs in panel (**c**,**h**). Panel (**d**,**i**) shows the gating rationale for CD133^+^ cells based on positive Hoechst situation. For the definition of cCSCs, CD133-positive cells were gated based on EpCAM^+^Hoechst^+^ cells as illustrated in panel (**e**,**j**). The cell count of CD133^+^EpCAM^+^Hoechst^+^ after negative selection strategy was 17 cells (**e**) and 220 cells (**j**).

**Figure 4:**
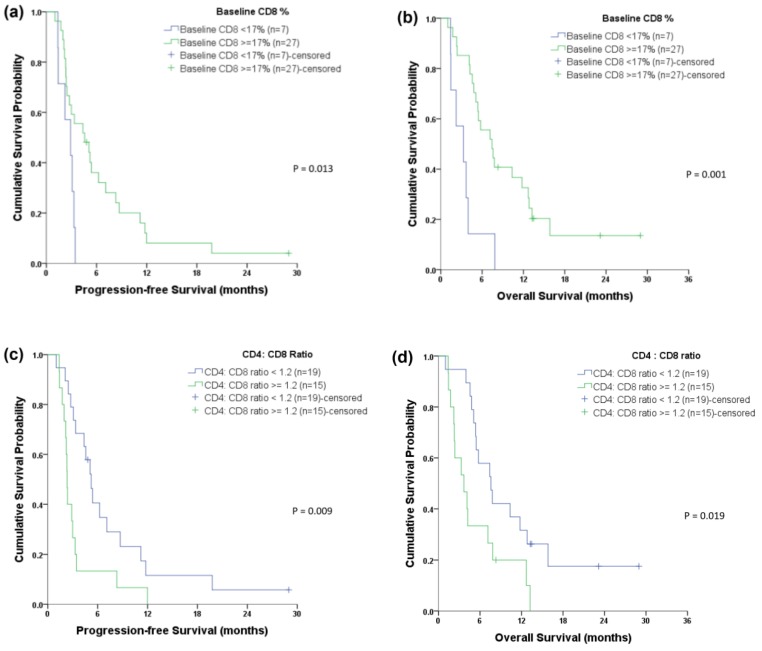
Kaplan-Meier curves for the survival analyses. A higher baseline CD8^+^ proportion was associated with better (**a**) progression-free survival (PFS) and (**b**) overall survival (OS). A lower baseline CD4: CD8 ratio was associated with better (**c**) PFS and (**d**) OS.

The CTC and cCSC analyses were performed using both cell line controls and samples from patients with HNSCC (Figure 2 and Figure 3). The lymphocytes’ PD-1 expression status is shown in Figure S1, as well as for the white blood cells (WBC) controls that underwent polyhydroxyalkanoate induction. Multivariate analyses revealed that disease progression was independently predicted by the baseline CTC number (hazard ratio [HR]: 1.01, 95% confidence interval [CI]: 1.005–1.022) and the cCSC ratio (HR: 10.92, 95% CI: 2.295–51.957) (Table 2). The multivariate analyses revealed that overall survival (OS) was independently predicted by the baseline CTC number (HR: 1.01, 95% CI: 1.003–1.017), the cCSC ratio (HR: 29.90, 95% CI: 5.420–164.992), and the baseline CD8^+^ proportion (HR: 0.24, 95% CI: 0.091–0.640). The Kaplan-Meier survival curves revealed that a high baseline CD8^+^ proportion (≥17%) predicted prolonged PFS and OS (Figure 4a,b), while a higher CD4: CD8 ratio predicted shorter Progression-free survival (PFS) and OS (Figure 4c,d). The details of CTC, cCSC, CD4, CD8, and CD4:8 ratios before and during chemotherapy were shown in Table S1. Their correlations among basic characteristics were listed in Table S2.

### 2.3. The cCSC Ratio May Contribute to Chemoresistance

**Figure 5:**
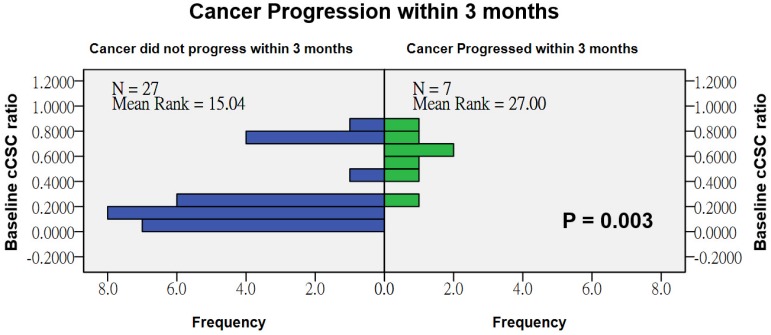
The presence of circulating cancer stem-like cells (cCSCs) could predict rapid disease progression within 3 months (*p* = 0.003 based on the Mann-Whitney *U* test).

We had hypothesized that a high cCSC ratio might predict a poor response to chemotherapy, based on the chemoresistance of cCSCs. Figure 5 and Table 2 show that a higher baseline cCSC ratio predicted disease progression within the first three months of chemotherapy (*p* = 0.003 based on the Mann-Whitney U test).

### 2.4. PD-1 Expression on CD4^+^, CD8^+^, and CD56^+^ T Lymphocytes

There were no significant associations between the survival outcomes and PD-1 expression on CD4^+^, CD8^+^, or CD56^+^ T lymphocytes.

## 3. Discussion

This prospective study evaluated 34 patients with initially unresectable, recurrent/metastatic HNSCC to examine whether chemotherapy response could be predicted using CTC, cCSC, and peripheral lymphocyte expression of PD-1. The results indicate that CTCs and cCSCs were independent predictors of cancer progression and OS and that patients with a high baseline CD8^+^ proportion had longer OS, while patients with a lower CD4: CD8 ratio had longer PFS. However, PD-1 expression on peripheral lymphocytes was not a significant predictor of the survival outcomes. To the best of our knowledge, this is the first study to examine CTC/cCSC and immune status using a single blood sample from patients with HNSCC.

Many studies have addressed the role of CTCs in HNSCC [25,26,27,28,29,30], including our previous study [8]. The results of the present study support the findings of the previous studies and indicate that CTC independently predicts prognosis in cases of HNSCC. In a previous stud [8], we cannot identify the prognostic role from CTC alone. One of the plausible reasons might be the improved stability and internal controls for the platform, which makes the enumerated CTC number more clinically and biologically significance by exclusion false positive signal. In addition, several studies have examined the role of cCSCs in cancer cases, which were examined using RT-PCR [31,32], counting with a gradient protocol [17,33], or flow cytometry [34,35]. Those results indicate that cCSCs are linked to a poor prognosis in cases of colorectal cancer [31,36], gastric cancer [17], prostate cancer [37], lung cancer [34,38], and hepatocellular carcinoma [32]. However, to the best of our knowledge, this is the first report to indicate that cCSCs reflect a positive prognosis in cases of HNSCC. In contrast, Park et al. [39] used a similar methodology and reported that the presence of CD133^+^EpCAM^+^ cells was associated with a poor prognosis in 10 cases of colorectal cancer. It is possible that the small sample sizes and different cancer types may explain the discrepancy between our findings and those of Park et al. Furthermore, studies have used different definitions of cCSCs, which we defined as expressing both CD133 and EpCAM after CD45 depletion. Thus, the different definitions might also explain the conflicting findings regarding the roles of cCSCs. For example, Toyoshima et al. defined cCSCs as CD44^+^ cells and reported that they had tumorigenicity [17], while Rentala et al. [40] defined cCSCs as cells that were CD133^+^CD44^+^. The most commonly used marker for identifying cCSCs is likely CD133 [31], and we included this marker to improve the ability to compare and validate our findings.

The present study revealed that high baseline peripheral CD8^+^ cells and low CD4: CD8 ratio were associated with a good prognosis. However, there is debate regarding the usefulness of peripheral lymphocytes versus tumor-infiltrating lymphocytes (TILs), as some investigators believe that TILs reflect the true tumor microenvironment and are correlated with prognosis and treatment response, despite the difficulty of repeated biopsy [41,42]. However, other reports have indicated that CD8^+^ TILs have no prognostic value in cases of HNSCC [39]. Therefore, we selected peripheral lymphocytes rather than TILs for the present study, as they are easier to monitor and may be useful as a follow-up marker (we hope to publish follow-up data in a later report).

Our results support the positive effects of higher baseline peripheral CD8^+^ T-cell proportions [40,43] and lower CD4:8 ratios [44,45] in cancer cases, especially among patients with HNSCC [46]. Nevertheless, our results are different from those reported by Dewyer et al., who found that pre-induction chemotherapy CD4^+^ T-cells but not CD8^+^ T-cells were correlated with a good prognosis and treatment response [47]. These differences may be related to the fact that we mainly enrolled patients with oral cavity cancer, while Dewyer et al. enrolled patients with laryngeal cancer, which suggests that the primary site may influence the prognostic markers. In addition, we examined the relative proportion of CD8^+^ cells, while Dewyer et al. performed absolute counting of lymphocytes. Thus, the different measurement units may explain the different findings.

The expression of PD-1 on lymphocytes has been widely discussed during recent years, and Waki et al. have reported that PD-1 expression on CD4^+^ and CD8^+^ T lymphocytes was correlated with OS among patients with lung cancer who received personalized peptide vaccination [22]. However, our study failed to detect significant relationships between the survival outcomes and the expression of PD-1 on CD4^+^, CD8^+^, or CD56^+^ T lymphocytes. It is possible that our negative findings are related to the small sample size and/or an insufficient cut-off for PD-1 positivity. For example, it may be more appropriate to use the difference between PD-1 staining and polyhydroxyalkanoate induction of control cells (Figure S1), and further adjustment of PD-1 staining or methodology standardization should be considered in future studies. However, Lyford-Pike et al. found that PD-1 expression on CD4^+^ T-cells and CD8^+^ T-cells was higher in tonsil tissue, compared to the peripheral blood, of patients with HNSCC [48], which indicates that PD-1 expression on peripheral blood lymphocytes may not be clinically significant, and our results support that possibility. Thus, PD-1 expression may only have prognostic value when it is observed on TILs.

The present study has several limitations. First, the sample size was small and a larger prospective study is needed to validate our findings. Second, despite the benefits of dynamic follow-up using peripheral lymphocytes, there is debate regarding whether they reflect the whole body’s immune status, as their properties are easily disturbed. Third, although we used a clear and strict definition for cCSCs, we did not isolate these cells and confirm that they possessed in-vivo tumorigenicity, invasion, or self-renewal, which is why we referred to them as “stem-like” cells.

## 4. Materials and Methods

### 4.1. Study Design

This prospective observational study examined baseline CTCs, cCSCs, and PD-1 expression on peripheral lymphocytes, as well as progression-free survival (PFS) and overall survival (OS) among patients with initially unresectable, recurrent/metastatic HNSCC. The analyses were performed when more than half of the events had occurred, and the ratio of cCSC to CTC was selected to evaluate its ability to predict cancer progression and cancer-related death. Two CTC samplings were performed, with the first being performed at baseline (seven days before the first dose of chemotherapy) and the second being performed at 14–28 days after the first dose of chemotherapy. Study results were reported according to the Reporting Recommendations for Tumour Marker Prognostic Studies (REMARK) guidelines.

### 4.2. Patient Enrollment

Patients were enrolled at the Chang Gung Memorial Hospital (Linkou, Taiwan), and the observational protocol was approved by the institutional review board of Chang Gung Memorial Hospital (103-5322B). All patients provided written informed consent before being enrolled. Patients were considered eligible if they had: (1) histologically or cytopathologically confirmed stage IVb HNSCC (initially unresectable or recurrent) or stage IVc HNSCC (metastatic), based on the 7th edition of the American Joint Committee on Cancer criteria, (2) were ≥20 years old, (3) provided informed consent, and (4) had adequate liver and renal functions with a sufficient white blood cell count (WBC) to tolerate the anticancer therapies (especially chemotherapy). Patients were excluded if they had synchronous cancer or previous cancers within the last five years.

Blood samples were drawn within seven days before the first dose of chemotherapy. Disease staging, disease management, and treatment response evaluations followed the standard treatment protocols and institutional guidelines and were based on the findings from computed tomography, pan-endoscopy, magnetic resonance imaging, and positron emission tomography. Chemotherapy was scheduled and delivered by medical oncologists who were part of the head and neck tumor board at Chang Gung Memorial Hospital. Based on the Response Evaluation Criteria in Solid Tumors guidelines (version 1.0), treatment response was classified as complete remission, partial response, stable disease, or progressive disease. Disease-specific PFS was calculated from the date of the first CTC sampling to the first instance of cancer-specific disease progression or death, and OS was calculated from the date of the first CTC sampling to the date of death from any cause.

### 4.3. Chemotherapy Regimens

Palliative chemotherapy regimens were selected after full discussions with the patient’s family. The regimens mainly contained cetuximab (400 mg/m^2^ and then 250 mg/m^2^ weekly for three consecutive months; Merck, Darmstadt, Germany), cisplatin (50–75 mg/m^2^ biweekly to triweekly), and 5-fluorouracil (700–1000 mg/m^2^/day as a continuous infusion during days 1–4 every 28 days). Other treatments option involved methotrexate (40 mg/m^2^ or a fixed weekly dose of 50 mg), bleomycin (15 mg weekly), and oral tegafur-uracil (300 mg/m^2^/day; TTY Biopharm Co. Ltd., Taipei, Taiwan) according to institutional guideline at Chang Gung Memorial Hospital.

### 4.4. Identifying CTCs and cCSCs

The CTCs were identified using negative selection and positive detection strategies, which were validated in our previous studies [49]. The first step involves a negative selection protocol that depletes red blood cells using lysis and leukocytes using a CD45 depletion kit (STEMCELL Technologies Inc., Vancouver, BC, Canada). The second step involves using flow cytometry to quantitatively identify CTCs (EpCAM^+^Hochest^+^CD45^−^) and cCSCs (CD133^+^EpCAM^+^Hochest^+^CD45^−^). The ratio of cCSCs to CTCs was calculated and reported as a percentage.

The CTC tests were performed using 4 mL of peripheral blood after discarding the first 4 mL of blood to avoid epithelial contamination. Red blood cell lysis was performed within 72 h, and further negative selection was performed using the EasySep CD45 Depletion Cocktail (25 μL/mL; STEMCELL Technologies Inc., Vancouver, BC, Canada) and EasySep Magnetic Nanoparticles (50 μL/mL; STEMCELL). The immunomagnetically enriched samples were subsequently spiked with OECM1 cells, labelled using an Alexa Fluor^®^ 488-conjugated monoclonal antibody to EpCAM (1:400; Cell Signaling Technology Inc., Danvers, MA, USA), and stained with Hoechst 33342 (1:500 in washing solution; Thermo Scientific, Waltham, MA, USA) for the nuclear staining. An isotype control antibody was used as an internal control, as well as peripheral blood samples from healthy individuals (4 mL) that were and were not spiked with 1000 OECM1 cells, which had been purchased from Taiwan’s Food Industry Research and Development Institute (Hsinchu, Taiwan). Performance recovery was defined as the proportion of OECM1 cells detected using flow cytometry (BD FACSCalibur; BD Biosciences, San Jose, CA, USA) to the number of spiked OECM1 cells, and a stable coefficient of variation (CV) value has been calculated in a previous report [49,50]. To be brief, the platform can have a recovery rate of 44.6 ± 9.1% and a % coefficient of variation (CV) of 20.4%. The previous platform reported in 2015 would detect 13.1 ± 0.9 cells/mL in healthy individuals (*n* = 20) [49], which was confusing and might be background signal (false positive). In this revised platform, isotype control was used for each sample, which resulted in a range of 0.0–3.0 cells/mL in healthy individuals in this study cohort (*n* = 20). CTCs were defined as cells that were positive for both EpCAM and Draq5. Immunohistochemistry was performed to detect p16 using a prediluted mouse monoclonal antibody (CINtec p16INK4a [clone E6H4]; Roche Laboratories Inc., Westborough, MA, USA) [51] and a Dako Autostainer (Dako North America Inc., Carpinteria, CA, USA), according to the manufacturer’s protocol.

### 4.5. PD-1 Staining and Controls

Peripheral blood mononuclear cells were isolated from 4 mL of healthy volunteers’ blood using a Ficoll-Hypaque density gradient. The cells were then cultured in RPMI medium containing 10% fetal bovine serum (FBS) for 72 h with or without stimulation using 2% phytohemagglutinin (M form; Thermo Fisher GIBCO, Waltham, MA, USA.) (Figure S1). The harvested cells were subsequently used in the immunoassays as controls that were positive and negative for PD-1 expression.

### 4.6. Immune Activity Assay

After red blood cell lysis, WBCs were collected from 2 mL of the patients’ blood and subjected to flow cytometry after surface staining using a CD4-FITC antibody (eBiosciences; San Diego, CA, USA), a CD8a-PE-Cy7 antibody (eBiosciences), and a PD-1-PE antibody (BD Pharmingen, San Jose, CA, USA). A total of 2 × 10^5^ WBCs were resuspended in 100 μL of RPMI medium with 10% FBS, 0.25 µg of the CD4-FITC antibody, 0.06 µg of the CD8a-PE-Cy7 antibody, and 20 µL of the PD-1-PE antibody. The corresponding isotype control antibodies were used under the same conditions. After staining for 1 h, the cells were washed using 2 mL of phosphate-buffered saline solution containing 10% FBS and 1 mM EDTA. The fluorescence measurements were performed using a CytoFLEX Flow cytometer (Beckman Coulter, Brea, CA, USA). The gating strategies were (1) 2 × 10^4^ events for CD4^+^/CD8^+^-gated cells, (2) positive and negative signals for PD-1 expression were based on the CD4^+^ or CD8^+^ cursors to yield positive signals after subtracting the signal from the PE-conjugated isotype control, and (3) the percentages of CD4^+^ and CD8^+^ cells were calculated, as well as the proportions of CD4^+^ and CD8^+^ cells that were positive for PD-1.

### 4.7. Statistical Analysis

The patients’ demographic data were reported as number (percentage) for categorical variables, and median (range) for continuous variables. Univariate and multivariate analysis were used, all factors used in the univariate analysis were examined in multivariate analysis but only those factors with statistical significance were displayed. A multivariate Cox’s proportional hazard model using the forward stepwise approach was used to examine the variables’ associations with OS and PFS. A risk model was also developed from the multivariate analysis. Kaplan-Meier curves and the log-rank test were used to examine any differences in the OS and PFS outcomes and determine the cutoffs, including the ratio of CD8^+^ and CD4: CD8. All analyses were performed using SPSS software (version 18; SPSS Inc., Chicago, IL, USA), and *p*-values of <0.05 were considered statistically significant.

## 5. Conclusions

This prospective study revealed that baseline CTCs and the cCSC ratio (i.e., cancer status), as well as the CD8^+^ proportion and the CD4: CD8 ratio (i.e., host immune status), were significantly associated with the prognosis of HNSCC. Furthermore, the baseline cCSC ratio could predict chemotherapy response during the first three months, which suggests that these cells are involved in chemoresistance.

## Figures and Tables

**Table 1:** The patients’ characteristics.

Characteristic	*n*	%	Baseline CTCs (mean ± SD)	*p* ^a^
Age (median, range), years	50 (37–73)		56.0 ± 56.4	
Sex				
Male	29	85.3%	51.0 ± 52.4	
Female	5	14.7%	84.8 ± 76.2	0.252
Primary site				
Oral cavity	19	55.9%	55.9 ± 55.9	
Oropharynx	8	23.5%	54.0 ± 68.3	
Hypopharynx	5	14.7%	68.1 ± 56.3	
Larynx	1	2.9%	40.0	
Paranasal sinus	1	2.9%	29.8	0.974
p16 status *				
Positive	3	8.8%	72.4 ± 25.6	
Negative	8	23.5%	22.8 ± 13.2	
Not examined	23	67.6%	52.0 ± 10.8	0.306
Stage IVb/IVc (AJCC 7th edition)	4/30		46.6 ± 66.1/61.1 ± 51.3	0.482
Metastatic site (*n* = 30)				
Lung	16	53.3%	59.0 ± 14.8	
Distant lymph node or soft tissue metastasis	11	36.7%	37.5 ± 11.3	
Bone	11	36.7%	33.6 ± 10.1	
Skin carcinomatosis	9	30.0%	68.6 ± 22.9	
Liver	2	6.70%	30.9 ± 21.9	0.610
First-line palliative chemotherapy	34	100.0%		
Cisplatin-based therapy ± cetuximab	28	82.4%	63.1 ± 59.5	
Non-platinum regimens (cisplatin-refractory)	6	17.6%	22.8 ± 17.1	0.114

* p16 immunohistochemistry staining for p16 expression was examined in 11 patients, including eight oropharyngeal, three hypopharyngeal, and one laryngeal cancer patients. AJCC = American Joint Committee on Cancer. CTC = circulating tumor cells. ^a^. The P values were calculated by independent Mann-Whitney U tests.

**Table 2:** Univariate and multivariate analyses of survival.

Factor	PFS				OS			
	Univariate		Multivariate		Univariate		Multivariate	
	HR (95% CI)	*p*-Value	HR (95% CI)	*p*-Value	HR (95% CI)	*p*-Value	HR (95% CI)	*p*-Value
Age	1.001 (0.962–1.041)	0.968			1.005 (0.965–1.047)	0.805		
Primary site	1.059 (0.799–1.404)	0.688			0.986 (0.759–1.280)	0.913		
ECOG	1.341 (0.856–2.100)	0.200			1.571 (1.062–2.326)	0.024		
CTC	1.008 (1.000–1.015)	0.036	1.013 (1.005–1.022)	0.002	1.005 (0.999–1.010)	0.12	1.010 (1.003–1.017)	0.004
cCSC ratio	4.367 (1.206–15.808)	0.025	10.920 (2.295–51.957)	0.003	9.788 (2.300–41.661)	0.002	29.903 (5.420–164.992)	<0.0001
PD1 on CD4^+^	1.001 (0.985–1.017)	0.897			0.995 (0.977–1.013)	0.58		
PD1 on CD56^+^	1.013 (0.991–1.035)	0.235			1.001 (0.982–1.020)	0.918		
PD1 on CD8^+^	0.997 (0.971–1.024)	0.828			1.002 (0.979–1.025)	0.872		
CD4^+^	1.022 (0.991–1.054)	0.174			1.003 (0.975–1.031)	0.837		
CD56^+^	0.961 (0.922–1.002)	0.060			0.965 (0.922–1.010)	0.123		
CD8^+^ proportion ≥17% vs. <17%	0.319 (0.124–0.821)	0.018			0.236 (0.094–0.595)	0.002	0.242 (0.091–0.640)	0.004
CD4:CD8 ratio ≥1.2 vs. <1.2	2.538 (1.230–5.237)	0.012	2.120 (0.959–4.688)	0.064	2.403 (1.130–5.109)	0.023		

Abbreviations: ECOG PS, Eastern Cooperative Oncology Group performance status; HR, hazard ratio; PFS, progression-free survival; OS, overall survival; CTC, circulating tumor cells; cCSC, circulating cancer stem-like cells; cCSC ratio, CD133^+^EpCAM^+^Hoechst^+^CD45^−^ cells divided by EpCAM^+^Hoechst^+^CD45^−^ cells.

## Supplementary Materials

The following are available online at https://www.mdpi.com/2072-6694/11/4/540/s1, Figure S1: Phytohemagglutinin induction for PD-1 expression as controls and the PD-1 expression on CD4^+^ and CD8^+^ cells analysis using flow cytometry. Table S1: Circulating tumor cells, circulating cancer stem-like cells and peripheral lymphocytes with chemotherapy responses, Table S2. Correlations among basic characteristics and baseline cCSC, CD8% and CD4:8 ratio.
