# Supplementary material for: The Prognostic Roles of Pretreatment Circulating Tumor Cells, Circulating Cancer Stem-Like Cells, and Programmed Cell Death-1 Expression on Peripheral Lymphocytes in Patients with Initially Unresectable, Recurrent or Metastatic Head and Neck Cancer: An Exploratory Study of Three Biomarkers in One-time Blood Drawing
Source: Cancers (Basel). 2019 Apr 15;11(4):540. doi: 10.3390/cancers11040540 (PMC6521270; doi:10.3390/cancers11040540)
Supplement: Supplementary file 1 [file cancers-11-00540-s001.pdf]

cancers

MDPI

Supplementary Materials: The Prognostic Roles of Pretreatment Circulating Tumor Cells, Circulating Cancer Stem-Like Cells, and Programmed Cell Death-1 Expression on Peripheral Lymphocytes in Patients with Initially Unresectable, Recurrent or Metastatic Head and Neck Cancer: An Exploratory Study of Three Biomarkers in One-time Blood Drawing

Pei-Hung Chang, Min-Hsien Wu, Sen-Yu Liu, Hung-Ming Wang, Wen-Kuan Huang, Chun-Ta Liao, Tzu-Chen Yen, Shu-Hang Ng, Jen-Shi Chen, Yung-Chang Lin, Hung-Chih Lin and Jason Chia-Hsun Hsieh

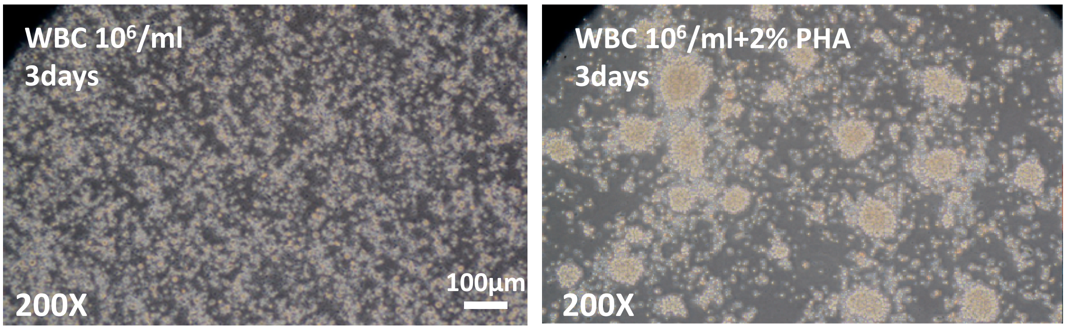

(a)

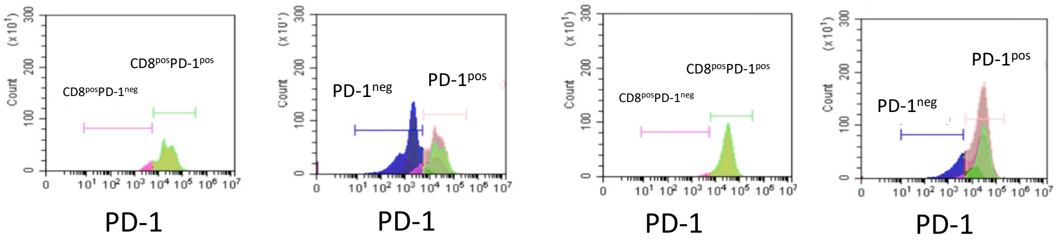

(b)

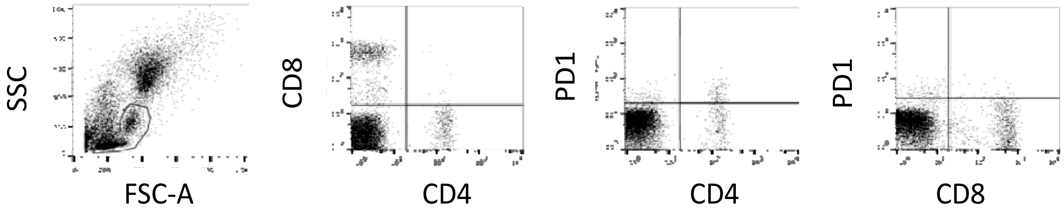

(c)

Figure S1. PD-1 expression controls. (**a**) Phytohemagglutinin induction, for (**b**) PD-1 expression as controls and (**c**) the PD-1 expression on CD4⁺ and CD8⁺ cells analysis using flow cytometry.

Table S1. Circulating tumor cells, circulating cancer stem-like cells and peripheral lymphocytes with chemotherapy responses.

ID	3-month Response	CTC-1	CTC-2	cCSC-1	cCSC-2	cCSC1 Ratio	cCSC-2 Ratio	CD4-1	CD4-2	CD8-1	CD8-2	CD56-1	CD56-2	CD4:8-1 Ratio	CD 4:8-2 Ratio
#1	PR/SD	43.00	76.50	6.50	34.50	0.1512	0.4510	10.50	9.50	20.50	18.30	1.23	1.50	0.5122	0.5191
#2	PR/SD	5.50	3.50	1.30	2.50	0.2364	0.7143	15.40	10.40	23.50	25.40	10.50	11.50	0.6553	0.4094
#3	PD	89.30	10.70	39.70	8.20	0.4446	0.7664	34.18	9.65	23.55	9.68	3.86	11.35	1.4514	0.9969
#4	PD	5.50	61.60	3.50	0.13	0.6364	0.0021	19.51	0.45	19.08	17.19	23.47	24.85	1.0225	0.0262
#5	PR/SD	20.00	125.50	15.50	25.25	0.7750	0.2012	10.40	18.76	23.64	26.54	1.65	1.56	0.4399	0.7069
#6	PR/SD	18.80	5.78	2.80	0.00	0.1489	0.0000	10.45	5.78	42.25	18.42	35.27	52.40	0.2473	0.3138
#7	PR/SD	71.50	85.50	8.50	9.50	0.1189	0.1111	7.03	7.50	77.15	25.34	1.16	1.01	0.0911	0.2960
#8	PR/SD	8.00	87.20	6.80	13.20	0.8500	0.1514	21.10	20.84	12.47	19.57	18.93	27.30	1.6921	1.0649
#9	PR/SD	10.75	3.25	2.75	2.50	0.2558	0.7692	21.71	24.50	18.33	40.56	13.87	7.52	1.1844	0.6040
#10	PR/SD	35.00	39.00	4.00	4.50	0.1143	0.1154	20.89	25.34	30.10	28.32	2.54	0.52	0.6940	0.8948
#11	PR/SD	158.50	223.50	9.75	8.00	0.0615	0.0358	30.50	23.20	35.60	40.30	15.30	19.50	0.8567	0.5757
#12	PR/SD	72.75	67.00	6.75	10.50	0.0928	0.1567	25.60	13.50	32.10	28.40	10.50	9.80	0.7975	0.4754
#13	PR/SD	40.00	58.75	10.25	9.25	0.2563	0.1574	42.40	32.40	40.10	26.50	2.50	2.10	1.0574	1.2226
#14	PD	29.75	245.50	20.50	80.25	0.6891	0.3269	18.74	40.65	13.65	28.65	1.34	0.56	1.3729	1.4188
#15	PR/SD	27.67	129.25	1.25	5.50	0.0452	0.0426	32.50	28.40	52.50	32.50	10.50	21.70	0.6190	0.8738
#16	PR/SD	5.50	2.00	2.25	0.00	0.4091	0.0000	22.50	25.40	18.40	45.50	20.50	21.60	1.2228	0.5582
#17	PR/SD	11.00	3.50	1.20	0.00	0.1091	0.0000	30.50	21.78	31.50	10.65	21.50	21.78	0.9683	2.0451
#18	PR/SD	10.50	131.50	8.25	80.10	0.7857	0.6091	18.63	28.13	20.58	36.51	10.16	9.21	0.9052	0.7705
#19	PR/SD	57.70	10.40	1.00	0.00	0.0173	0.0000	34.10	13.54	41.82	48.46	10.23	12.23	0.8154	0.2794
#20	PD	9.80	270.25	8.50	87.50	0.8673	0.3238	36.78	39.10	28.50	24.50	4.12	15.89	1.2905	1.5959
#21	PD	4.50	123.50	3.54	28.50	0.7867	0.2308	28.54	29.56	20.34	16.34	5.76	2.51	1.4031	1.8091
#22	PR/SD	45.25	43.50	32.25	28.50	0.7127	0.6552	24.23	10.43	18.23	8.12	12.55	8.10	1.3291	1.2845
#23	PR/SD	75.00	23.50	10.25	10.33	0.1367	0.4396	21.40	27.40	19.23	42.12	12.99	13.12	1.1128	0.6505
#24	PR/SD	47.50	46.50	10.25	11.50	0.2158	0.2473	25.25	10.54	10.21	9.50	21.16	25.90	2.4731	1.1095
#25	PR/SD	53.50	132.50	10.50	85.50	0.1963	0.6453	18.25	52.25	17.12	23.23	29.60	18.10	1.0660	2.2492
#26	PR/SD	96.50	32.50	75.50	15.25	0.7824	0.4692	51.00	25.30	32.38	10.23	2.54	2.51	1.5750	2.4731
#27	PR/SD	85.00	35.50	10.50	7.25	0.1235	0.2042	10.87	45.23	25.18	47.87	5.18	4.92	0.4317	0.9449
#28	PR/SD	215.50	5.60	10.00	1.00	0.0464	0.1786	52.32	25.75	32.98	26.57	10.23	25.12	1.5864	0.9691
#29	PR/SD	75.30	95.50	5.80	2.25	0.0770	0.0236	48.43	10.67	30.12	32.12	10.12	12.58	1.6079	0.3322
#30	PD	10.50	35.00	5.25	18.50	0.5000	0.5286	25.23	15.58	15.20	15.80	5.10	4.20	1.6599	0.9861
#31	PR/SD	7.50	1.50	1.50	1.00	0.2000	0.6667	40.25	30.15	20.56	45.86	1.50	10.50	1.9577	0.6574
#32	PR/SD	155.50	45.50	10.00	10.50	0.0643	0.2308	15.40	25.24	12.43	18.61	5.73	54.89	1.2389	1.3563
#33	PD	204.50	25.50	54.50	15.40	0.2665	0.6039	20.50	20.76	15.34	15.34	13.73	27.59	1.3364	1.3533
#34	PR/SD	96.40	117.25	27.25	35.25	0.2827	0.3006	10.76	40.50	10.12	42.54	2.12	0.00	1.0632	0.9520

Abbreviations: PR, partial response; SD, stable disease; PD, progressive disease; CTC, circulating tumor cells; cCSCs, circulating cancer stem-like cells.

Table S2. Correlations among basic characteristics and baseline cCSC, CD8% and CD4:8 ratio.

Factors	*n*	%	Baseline sCSC Ratio (Mean±SD)	*p*	Baseline CD8 % (Mean±SD)	*p*	Baseline CD4:8 Ratio (Mean±SD)	*p*
Age (median, range), years	50 (37–73)							
Sex								
Male	29	85.3%	0.35±0.30		26.43 ± 13.2		1.13 ± 0.51	
Female	5	14.7%	0.24 ± 0.17	0.436	23.67 ± 17.02	0.681	1.02 ± 0.50	0.679
Primary site								
Oral cavity	19	55.9%	0.33 ± 0.29		24.79 ± 10.84		1.09 ± 0.53	
Oropharynx	8	23.5%	0.69±0.00		13.65 ± 0.00		1.37 ± 0.00	
Hypopharynx	5	14.7%	0.43±0.32		23.85 ± 9.58		1.29 ± 0.48	
Larynx	1	2.9%	0.16 ± 0.14		34.04 ± 25.44		0.81 ± 0.47	
Paranasal sinus	1	2.9%	0.26 ± 0.00	0.361	40.10 ± 0.00	0.418	1.06 ± 0.00	0.537
p16 status								
Positive	3	8.8%	0.22 ± 0.19		9.49 ± 5.48		1.10 ± 0.74	
Negative	8	23.5%	0.42 ± 0.36		8.10 ± 2.86		1.29 ± 0.35	
Not examined	23	67.6%	0.32 ± 0.27	0.584	15.12 ± 3.15	0.356	1.05 ± 0.52	0.504
Stage IVb/IVc (AJCC 7th edition)	4/30		0.28 ± 0.22/0.37 ± 0.31	0.406	26.89 ± 17.67/25.55 ± 11.15	0.788	0.91 ± 0.53/1.22 ± 0.46	0.081
Metastatic site (*n* = 30)								
Lung	16	53.3%	12.69 ± 19.01		28.41 ± 11.32		1.27 ± 0.53	
Distant lymph node or soft tissue metastasis	11	36.7%	19.02 ± 23.62		27.00 ± 13.46		1.02 ± 0.41	
Bone	11	36.7%	14.33 ± 20.94		27.90 ± 11.72		1.23 ± 0.48	
Skin carcinomatosis	9	30.0%	17.67 ± 17.46		20.05 ± 6.75		0.89 ± 0.39	
Liver	2	6.70%	9.50 ± 1.41	0.914	22.81 ± 8.05	0.442	1.18 ± 0.16	0.361
First-line palliative chemotherapy	34	100.0%						
Cisplatin-based therapy ± cetuximab	28	82.4%	13.43 ± 17.52		27.38 ± 14.19		1.03 ± 0.46	
Non-platinum regimens (cisplatin-refractory)	6	17.6%	8.67 ± 6.36	0.521	19.67 ± 8.16	0.210	1.48 ± 0.60	0.044

Abbreviations: CTC, circulating tumor cells; cCSCs, circulating cancer stem-like cells.

© 2019 by the authors. Submitted for possible open access publication under the terms and conditions of the Creative Commons Attribution (CC BY) license (http://creativecommons.org/licenses/by/4.0/).
